# Derivation of Corneal Keratocyte-Like Cells from Human Induced Pluripotent Stem Cells

**DOI:** 10.1371/journal.pone.0165464

**Published:** 2016-10-28

**Authors:** Richard W. Naylor, Charles N. J. McGhee, Chad A. Cowan, Alan J. Davidson, Teresa M. Holm, Trevor Sherwin

**Affiliations:** 1 Department of Ophthalmology, University of Auckland, Auckland 1142, New Zealand; 2 Harvard Stem Cell Institute, Harvard University, Cambridge, Massachusetts 02138, United States of America; 3 Department of Molecular Medicine and Pathology, University of Auckland, Auckland 1142, New Zealand; Cedars-Sinai Medical Center, UNITED STATES

## Abstract

Corneal diseases such as keratoconus represent a relatively common disorder in the human population. However, treatment is restricted to corneal transplantation, which only occurs in the most advanced cases. Cell based therapies may offer an alternative approach given that the eye is amenable to such treatments and corneal diseases like keratoconus have been associated specifically with the death of corneal keratocytes. The ability to generate corneal keratocytes *in vitro* may enable a cell-based therapy to treat patients with keratoconus. Human induced pluripotent stem cells (hiPSCs) offer an abundant supply of cells from which any cell in the body can be derived. In the present study, hiPSCs were successfully differentiated into neural crest cells (NCCs), the embryonic precursor to keratocytes, and then cultured on cadaveric corneal tissue to promote keratocyte differentiation. The hiPSC-derived NCCs were found to migrate into the corneal stroma where they acquired a keratocyte-like morphology and an expression profile similar to corneal keratocytes *in vivo*. These results indicate that hiPSCs can be used to generate corneal keratocytes *in vitro* and lay the foundation for using these cells in cornea cell-based therapies.

## Introduction

The cornea represents the transparent anterior-most portion of the eye. It acts to protect the underlying iris, pupil and anterior chamber as well as providing two-thirds of the refractive power of the eye. A healthy cornea has a central thickness of about 490 to 620 μm, 90% of which consists of the stromal layer. The corneal stroma is composed of highly organised collagen fibrils which arrange into lamellae that run parallel to the corneal surface [1]. The corneal stroma is populated by a small number of non-myelinated nerve bundles and trafficking immune cells, but its main cellular occupant is the corneal keratocyte.

Corneal keratocytes are derived from neural crest cells (NCCs). During embryogenesis, NCCs occupy the presumptive cornea at around E10.5 in mice and subsequently differentiate into keratocytes, causing thickening of the stroma [2, 3]. Keratocytes are quiescent, mesenchymal-like cells which extend out keratopodia that contact neighbouring keratocytes, forming a continuously linked cell population within the stroma [4]. Keratocan and Lumican are important keratan sulphate-containing proteoglycans that are highly expressed in corneal keratocytes [5] and regulate transparency of the cornea by organising and maintaining the topography of collagen fibrils so as to minimise ocular opacity [6]. When this function is perturbed, corneal health and transparency is affected [7]. In patients suffering from keratoconus, there is a reduction in the number of corneal keratocytes in the stroma due to apoptosis [8]. This loss, together with reduced thickness of the stroma, leads to ectasia that is characterised by a conical cornea due to its thinning and protrusion [9–11]. In patients with advanced keratoconus, corneal scarring may also be present [12]. Corneal scarring is associated with activated keratocytes responding to a pathological environment, and their conversion to myofibroblasts that deposit non-transparent fibrotic tissue [13]. The definitive treatment available to patients with advanced keratoconus is corneal transplantation, a procedure that depends on donor tissue availability and may be complicated by immunological rejection and graft failure.

Given the pathophysiology of keratoconus is believed to mainly be associated with loss of corneal keratocytes, it remains possible that alternative, cell-based therapies could be adopted to reduce dependency on donor tissue. The discovery that adult somatic cells retain the ability to be reprogrammed into pluripotent cells [14, 15] has provided a limitless resource of stem cells from which any cell type in the body can be attained. Human induced Pluripotent Stem Cells (hiPSCs) have been used to generate many cell types, such as retinal pigment epithelium [16, 17], which is currently being utilised in clinical trials for autologous transplant into patients with age-related macular degeneration [18]. The use of hiPSCs is ethically less problematic than human embryonic stem cells and hiPSCs can be generated from the patient, reprogrammed into another cell type and used for autologous transplantation, reducing the risk of immunological rejection.

This study establishes an *in vitro* protocol for differentiating hiPSCs into cells with a corneal keratocyte-like phenotype. hiPSCs were first differentiated into NCCs before being introduced to two different downstream culturing methods. In the first method, hiPSC-derived NCCs were cultured in 3D and a keratocyte-like fate was adopted, these cells expressed and deposited Keratocan. However, qPCR analysis of these cells suggested they were dissimilar to *in vivo* corneal keratocytes. In the second method, hiPSC-derived NCCs were exposed to cadaveric corneal tissue where they actively migrated between the stromal collagen fibrils and showed morphological as well as transcriptional similarity to corneal keratocytes *in vivo*.

## Materials and Methods

### Tissue Culture

Tissue samples and cell lines were prepared and used in accordance with Health and Disability Ethics Committee approvals assigned by the Ministry of Health New Zealand (ref# NTX/07/08/080/AM04). All cultures were grown in a 37°C, 5% CO_2_ humidified incubator. hiPSCs were grown on Geltrex (ThermoFisher) in Modified Tennelle’s Special Recipe 1 (MTESR1, StemCell Technologies). All cells were dissociated and passaged to a new well/ plate or into a single cell suspension using Accutase (StemCell Technologies). The iPSC line used was generated using modified RNAs encoding the Yamanaka factors and was first described by Warren *et al*, (2010) [19].

### NCC production

We followed the protocols outlined in Chambers *et al* (2013), and Lee *et al* (2010) [19, 20]. To begin, a confluent 10 cm cell culture dish was passaged to Geltrex coated 6-well plates (NCCs were not grown on mitomycin treated mouse embryonic fibroblasts). The number of cells was estimated using a haemocytometer and transferred to a 6-well plate at a concentration of 10, 000–20, 000 cells/ cm^2^. 10 μM Y-27632 was used in the media for the first 24 hours after passaging only. Due to growth, cells were split 1:2 once during each protocol, typically between day 6 and day 8, using Accutase (StemCell Technologies). Upon completion of NCC derivation, cells were frozen back for storage in liquid nitrogen (in 10% DMSO/ 90% BSA) or used for downstream applications. We repeated this protocol a total of eleven times on different passages of the iPSCs and consistently yielded >80% NCC conversion.

### 3D culturing

The cells of one well of a 6-well plate (~2 x 10^5^ cells) were dissociated and made into a single-cell suspension in a 15 ml Falcon tube using Accutase. These cells were then pelleted at 700 g for 5 mins. The media was aspirated and carefully replaced with Keratocyte Differentiation Media (Advanced DMEM (Life Technologies), 10 ng/ml FGF2, 0.1 mM ascorbic acid-2-phosphate). Pelleted cells were left to grow for 21 days with media changes every second day.

### Limbal rim assay

Corneo-scleral Limbal rims were freeze thawed (20 mins at -80°C, 20 mins in a 37°C water bath x10) to degrade any endogenous protein epitopes. Rims were cut into slices using a scalpel and each slice was placed in one well of a 12-well plate. 1.5 ml of KDM was added to each well. hiPSC-derived NCCs were dissociated from one well of a 6-well plate and placed in a 15 ml Falcon tube. Cells were then pelleted at 700g for 5 minutes. Media was aspirated from the 15 ml Falcon tube and the cells were re-suspended in 8 ml of KDM. Cell number was determined using a haemocytometer and ~2 x 10^5^ cells were seeded directly onto the sclera region of each limbal rim slice using a Hamilton syringe. We seeded cells on the sclera as we found the conical shape of the cornea caused the unattached cells to accumulate at the region of the limbal rim. The following day, live/ dead cell staining was performed using LIVE/DEAD Viability/Cytotoxicity Kit for mammalian cells (Molecular Probes), to detect if cells had appropriately attached to the rim.

### Cryosectioning

3D cultured NCCs or limbal rim slices seeded with NCCs were washed twice in PBS and fixed in 4% PFA overnight at 4°C. The tissues were washed twice in PBS and then placed in 1% low melting point agarose containing 5% sucrose and 0.9% agar (made up in water) in cryomoulds. After one hour the cryoblocks were removed from the cryomoulds and placed in 30% Sucrose/PBS. Cryoblocks were incubated overnight at 4°C then 30% Sucrose/PBS was replaced and left for another night at 4°C. Cryoblocks were flash frozen on dry ice and stored at -80°C prior to sectioning. 14 μm transverse sections were cut in a cryostat machine (set to -25°C) and transferred to a gelatine-coated microscope slide.

### Immunohistochemistry

Slides containing cryosections were dried overnight at 4°C and then washed twice in Tris Buffered Saline containing 0.1% Triton X100 (TBST). Slides were then placed in block solution (3% BSA, 5% Goat serum in TBST) for at least one hour. The primary antibody was then applied in the same block solution (all at 1:100) and left overnight at 4°C; SOX2 (R&D Systems, MAB2018), AP2a (DSHB, 3B5), p75^NTR^ (Advanced Targeting Systems, AB-N07) Keratocan (SantaCruz, SC-66941), HNK1 (SigmaAldrich, C6680), Vimentin (Abcam, ab92547) and ABCB5 (SigmaAldrich, HPA026975) were used. Slides were washed 4 x 15 mins in TBST and incubated in secondary antibody (Abcam, 96901 and 96883) at 1:500 in TBST for 2 hours at room temperature. Slides were then washed twice with TBST and mounted under a coverslip with Prolong Gold (ThermoFisher).

### Quantitative Real-Time PCR

Cultures of hiPSCs and NCCs in 6-well plates were washed twice in PBS and then exposed to 1 ml of Trizol for 10 mins. Trizol extracted RNA was then transferred to a 1.5 ml eppendorff tube and stored at -80°C. 3D-cultured NCCs and limbal rim slices were similarly treated with 1 ml of Trizol and transferred to a flat-bottomed 2 ml eppendorff tube. The tissue was then homogenised and stored at -80°C. RNA was purified from Trizol using a PureLink^TM^ RNA Mini Kit (Ambion) and cDNA was prepared using a PrimeScript^TM^ RT Master Mix (Takara). Each reaction of real-time PCR used 8 μl of a master mix containing SYBR® Premix Ex Taq (Tli RnaseH Plus), ROX plus (Takara) and 10 μM primer mix, and 2 μl of diluted cDNA. Primers for SYBR assays were synthesized using the Harvard PrimerBank as a reference. All amplifications were performed in triplicate, with *HPRT1* used for normalization of RNA content. A negative control with no cDNA was also used in each qPCR. Relative expression was determined using the comparative C_T_ method (2^-∆∆CT^).

## Results

### Differentiation of hiPSCs into NCCs

**Fig 1:**
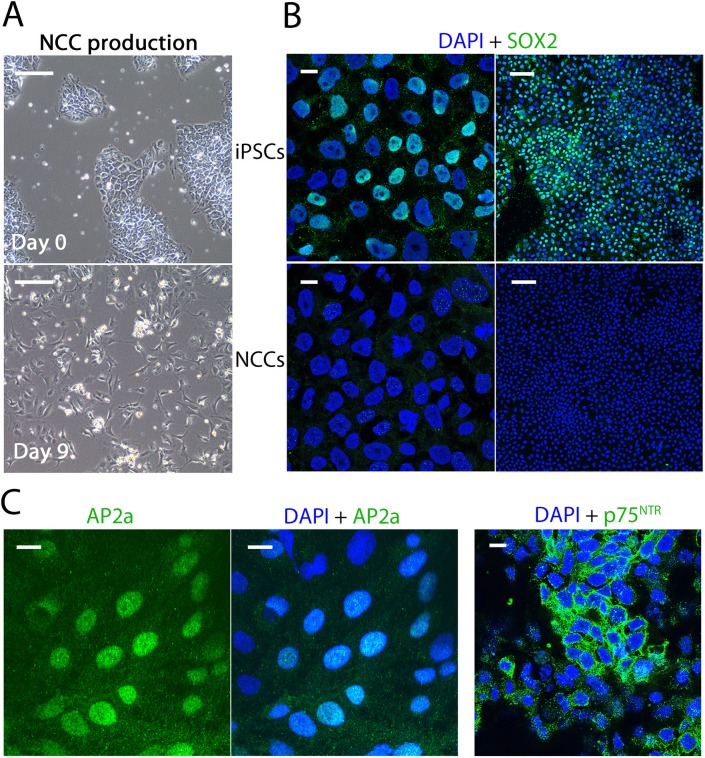
Generation of Neural Crest Cells (NCCs) from human induced Pluripotent Stem Cells (hiPSCs). (A) hiPSC colonies were observable at day 0 of a 12-day protocol for Neural Crest Cells (NCCs) derivation. On day 9, cells were more mesenchymal in appearance and possessed a typical NCC-like dendritic morphology. (B)(C) We observed hiPSCs were SOX2^+^, but hiPSC derived NCCs were SOX2^-^. >80% of derived NCCs were positive for neural crest markers AP2a and p27^NTR^. Brightfield scale bar represents 200 μm, low magnification (10X) confocal images scale bar represents 100 μm, high magnification (63X) confocal images scale bar represents 10 μm.

As corneal keratocytes are derived from cephalic NCCs during embryogenesis [2, 3] it was reasoned that a 2-step protocol could be developed whereby hiPSCs were first induced to differentiate into NCCs, of which a number of protocols have been developed [20–25], and then matured into keratocytes in the second step. Two established NCC protocols (Chambers *et al*, (2013) and Lee *et al*, (2010)) were performed and were compared for which worked most efficiently based on a number of variables; the percentage of NCCs made, the levels of NCC genes expressed, the degree of reduction in pluripotent marker gene expression, and the cell morphology observed. It was found that the protocol described by Chambers *et al* (2013) worked most efficiently, producing cells with a NCC-like dendritic morphology, loss of SOX2-positive immunostaining and a high percentage (>80%) of cells labelled with the cephalic NCC markers AP2a and p75^NTR^ (Fig 1 and S1 Fig). Quantitative PCR (qPCR) analysis showed that the Chambers *et al* (2013) protocol performed better at yielding higher expression levels of neural crest genes (such as *PAX3*, *SOX9*, *SOX10*, *ZIC1*, and *TFAP2A*) than the Lee *et al* (2010) protocol (S2 Fig). As a result, the Chambers *et al* (2013) protocol was used for further study and protocol development.

**Fig 2:**
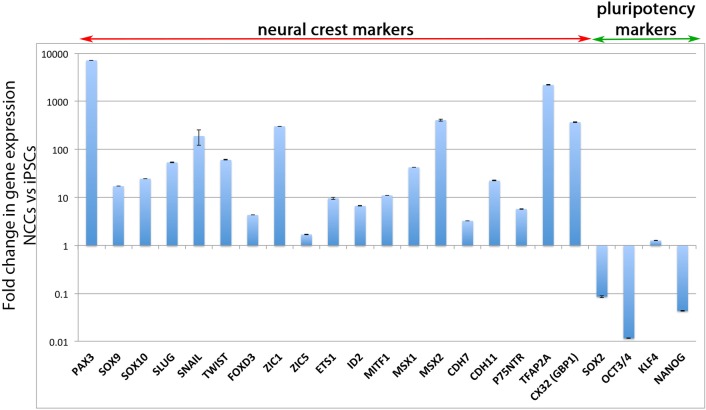
Expression analysis of derived NCCs compared to hiPSCs. Quantitative polymerase chain reaction (qPCR) analysis showed derived NCCs had higher expression of many neural crest markers relative to hiPSCs. The pluripotency markers *SOX2*, *OCT3/4* and *NANOG* were significantly reduced in NCCs, but *KLF4* expression was maintained at a similar level.

**Fig 3:**
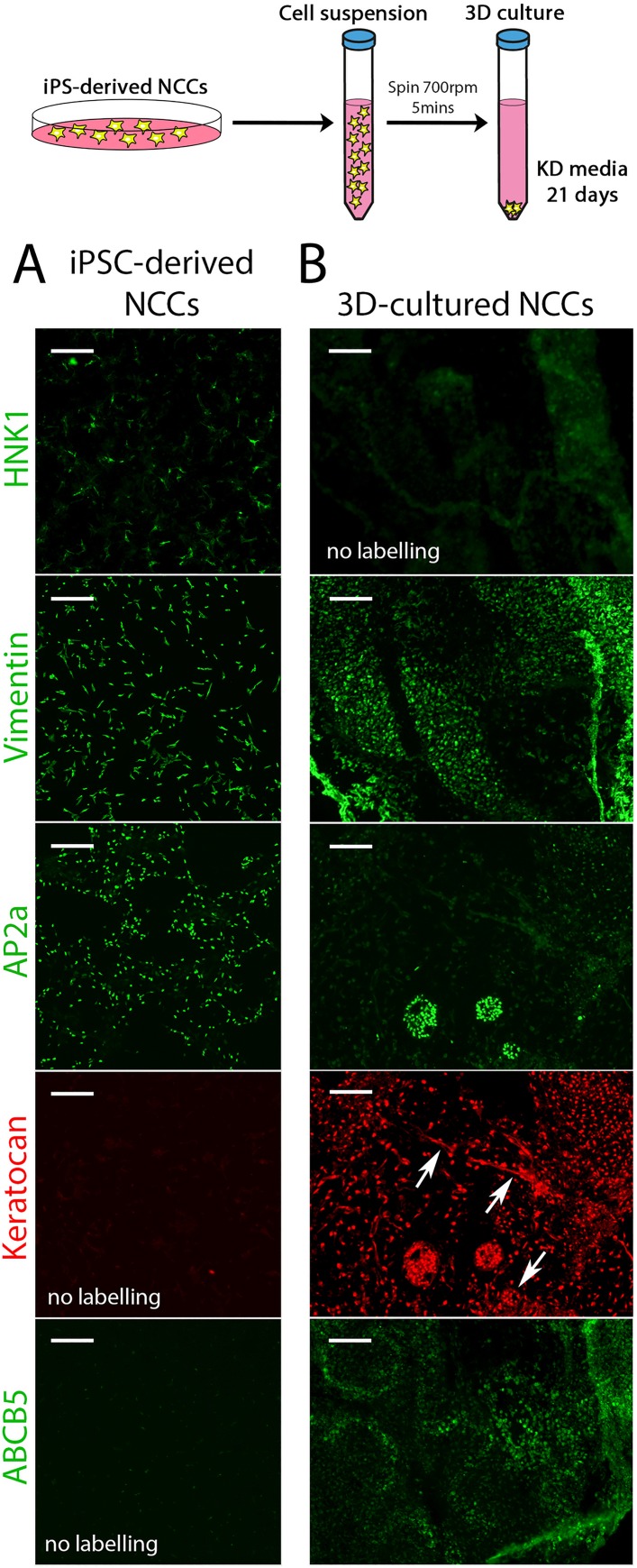
3D culturing promotes a corneal keratocyte-like cell fate. (A) Panels show hiPSC-derived NCCs stained for HNK1, Vimentin, AP2a, Keratocan (shown in red as it was co-stained with the AP2a panel) and ABCB5. (B) 3D cultures were generated as shown in the schematic at the top of the Figure. Panels show transverse cryosections of the 3D cultured NCCs stained for HNK1, Vimentin, AP2a, Keratocan (red) and ABCB5. Arrows in the Keratocan^+^ panel indicates extracellular deposits of Keratocan. Scale bar represents 100 μm.

Extensive qPCR analysis of NCC and pluripotency gene markers was undertaken to confirm the NCC identity of the hiPSC-derived NCCs. All neural crest markers tested were more highly expressed in the hiPSC-derived NCCs than in hiPSCs (Fig 2). In particular, *PAX3*, *SOX10*, *ZIC1*, *MSX2* and *TFAP2A* were >100-fold more highly expressed in the hiPSC-derived NCC population than in hiPSCs. The pluripotency genes *SOX2*, *OCT3*/*4* and *NANOG* were all reduced by at least 10-fold in hiPSC-derived NCCs compared to hiPSCs (Fig 2). *KLF4* expression did persist at a relatively unchanged level, which is consistent with *KLF4* being highly expressed in NCCs [26] and their derivatives (such as smooth muscle cells [27]), and with *KLF4* being the most highly expressed transcription factor in the mouse cornea [28]. In addition to analysing gene expression, we also undertook protein analysis (Fig 3A). We performed antibody labelling for HNK1, AP2a and Vimentin to test for NCC markers. All hiPSC-derived NCCs were positive for Vimentin and AP2a, and approximately 20% were positive for HNK1 (Fig 3A). These results, in addition to cell morphology observations, loss of SOX2 antibody staining and AP2a^+^ and p27^NTR+^ staining, support a conclusion whereby NCCs were successfully derived from hiPSCs.

### Culturing hiPSC-derived NCCs in 3D produces a corneal keratocyte phenotype

For the second step of our 2-step protocol, hiPSC-derived NCCs were further differentiated into cells with a keratocyte-like phenotype. Given that the corneal keratocyte exists within a collagenous matrix in the cornea stroma, we hypothesized that culturing NCCs in a 3D matrix may promote their conversion to a corneal keratocyte-like cell. To do this, a cell suspension of hiPSC-derived NCCs was pelleted and then cultured in a defined keratocyte differentiation media (see schematic in Fig 3 and ref [29]). After 21 days, this 3D culture had grown and did not dissociate upon agitation. These aggregates were fixed and cryo-sectioned for analysis by immuno-staining. Labelling for HNK1 and AP2a showed these markers were reduced after 3D-culture but for small patches of AP2a-positive staining (Fig 3B). Vimentin labelling persisted after 3D-culture, which is consistent with this protein being a label of corneal keratocytes as well as NCCs (Fig 3B). Keratocan and ABCB5 are specific markers of corneal keratocytes and were not detectable by protein analysis in hiPSC-derived NCCs (Fig 3A). Strikingly, all cells in the 3D-culture were positive for Keratocan and extracellularly deposited Keratocan could be observed (Fig 3B). ABCB5 staining was detected in approximately 30% of 3D-cultured cells (Fig 3B). These results suggest that hiPSC-derived NCCs cultured in 3D lose their neural crest identity and convert to a corneal keratocyte-like cell type.

**Fig 4:**
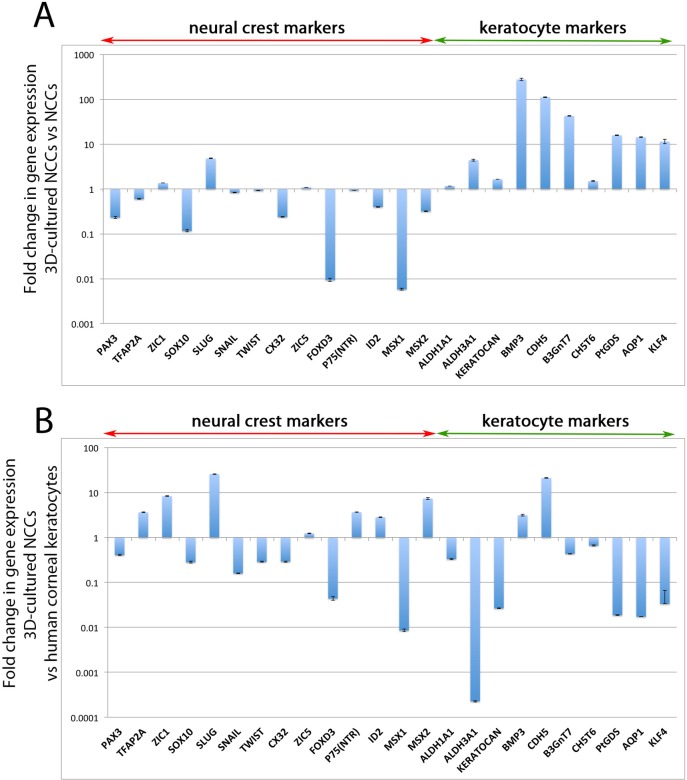
Expression analysis of 3D-cultured NCCs compared to non 3D-cultured NCCs and cadaveric human corneal keratocytes. (A) qPCR analysis showed 3D cultured derived NCCs had lower levels of expression of many neural crest markers, such as *PAX3*, *SOX10*, *CX32*, *FOXD3* and *MSX1*, compared to non-3D cultured NCCs. Genes that are highly expressed in corneal keratocytes were up-regulated by 3D culture. (B) The gene expression profile of 3D-cultured NCCs was largely incompatible with cadaveric corneal keratocytes. Most genes assayed had significantly higher or lower levels of gene expression when the two populations were compared.

To better analyse the potential fate change of the hiPSC-derived NCCs to a corneal keratocyte phenotype, we performed extensive qPCR analysis on the 3D-cultures. Relative to hiPSC-derived NCCs, there was a general down-regulation of NCC genes after the 3D-culture. *PAX3*, *SOX10*, *CX32*, *FOXD3* and *MSX1*, and to a lesser extent *TFAP2A*, *SNAIL*, *ID2* and *MSX2* had reduced levels of gene expression in 3D-cultured NCCs relative to hiPSC-derived NCCs (Fig 4A). A few neural crest genes increased their levels of expression when cultured in 3D, such as *ZIC1* and *SLUG*, but most of the assayed neural crest genes had reduced expression in hiPSC-derived NCCs cultured in 3D for 21 days (Fig 4A). Analysis of genes that are highly expressed in corneal keratocytes showed their expression levels increase after 3D-culture (Fig 4A). In particular, *BMP3*, *CDH5*, *B3GnT7*, *PtDGS*, *AQP1* and *KLF4* were >10-fold more highly expressed after 3D culturing (Fig 4A). *ALDH1A1*, *ALDH3A1*, *KERATOCAN* and *CHST6* were also increased. These results suggest that 3D culture of NCCs promotes conversion of these cells towards a keratocyte-like fate.

Subsequently, these *in vitro* derived corneal keratocyte-like cells were compared to *in vivo* human corneal keratocytes obtained from cadaveric corneas. RNA acquired from corneal buttons, stripped of their endothelial and epithelial layers, leaving only the stromal layer of the cornea, was used as a source of corneal keratocytes. qPCR analysis showed keratocyte markers *ALDH3A1*, *KERATOCAN*, *PtDGS*, *AQP1* and *KLF4* were expressed at much lower levels in 3D-cultured NCCs compared to human corneal keratocytes (Fig 4B). However, other corneal keratocyte markers were expressed at similar levels in both cell populations, including *ALDH1A1*, *BMP3*, *B3GnT7* and *CHST6* (Fig 4B). Neural crest genes *ZIC1*, *SLUG* and *MSX2* were more highly expressed in 3D-cultured NCCs relative to human corneal keratocytes whereas *SNAIL*, *FOXD3* and *MSX1* were more lowly expressed (Fig 4B). Despite this, a number of genes were expressed at similar levels in 3D-cultured NCCs relative to human corneal keratocytes, including *PAX3*, *SOX10* and *ZIC5* (Fig 4B).

In conclusion, the 3D culture of hiPSC-derived NCCs promotes both the expression of corneal keratocyte-specific genes and also the deposition of Keratocan in extracellular matrix suggesting that these cells can be induced to adopt a keratocyte fate *in vitro*.

### Culturing hiPSC-derived NCCs on limbal rims promotes keratocyte-like fate

**Fig 5:**
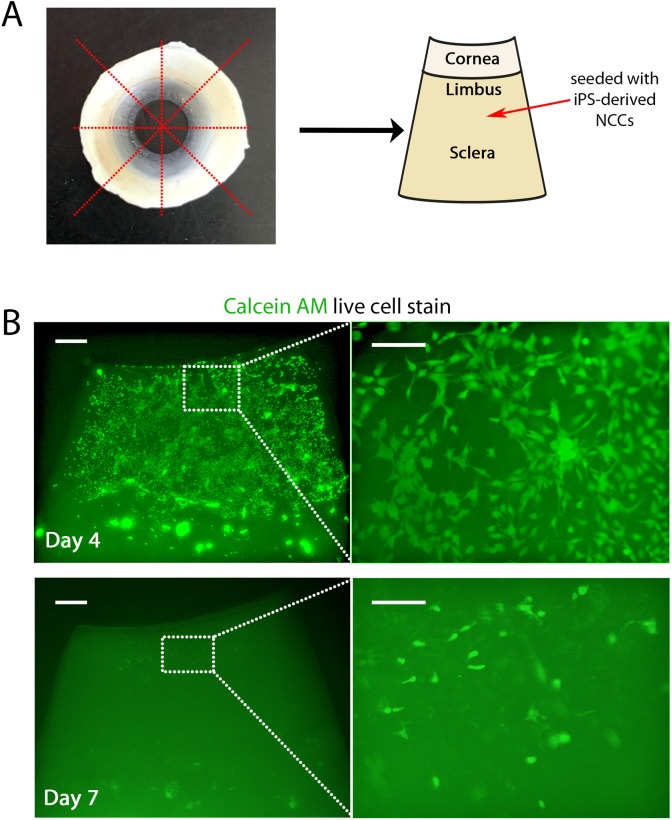
hiPSC derived NCCs seeded onto limbal rims migrate to the cornea and then migrate into the stroma. (A) hiPSC-derived NCCs were seeded onto the scleral region of limbal rim slices as shown in the schematic. (B) After 4 days, live cell staining with Calcein AM indicated all derived NCCs had migrated to the corneal surface region of the limbal rim slice. By day 7 derived NCCs had migrated into the stroma of the cornea. Scale bars for low magnification images (let hand side) represent 200 μm and for higher magnification images (right hand side) represent 100 μm.

Having demonstrated that a 3D-culture environment can induce keratocyte differentiation, it was next examined whether native corneal tissue would be more effective at inducing a keratocyte phenotype. To achieve this aim, hiPSC-derived NCCs were exposed to cadaveric corneal tissue. This technique used a donated, cadaveric, peripheral corneo-scleral rim (see Fig 5A), which was put through ten freeze-thaw cycles to deplete the cornea of endogenous proteins. Interestingly, before freeze-thaw cycling was performed, the limbal rims contained cells positive for AP2a and Keratocan (S3 Fig). AP2a is a marker of cephalic neural crest cells, therefore this result suggests corneal keratocytes still retain a marker of neural crest identity even in adult tissue.

To test if exposure to corneal tissue promoted conversion to a keratocyte-like fate, we seeded hiPSC-derived NCCs onto the sclera of a corneal rim slice (Fig 5A). These cells attached to the corneal slice and calcein AM live-cell staining showed they were still alive by day 4 (see S4 Fig for the dead cell stain) and had migrated to the corneal region of the rim (Fig 5B). Calcein AM imaging also showed that these cells displayed a NCC-like morphology and their movement towards the cornea indicated an intrinsic migratory capacity (Fig 5B). At day 7, NCCs on the surface of the cornea could no longer be observed (Fig 5B). Ethidium staining for dead cells showed no positive labelling (data not shown), but higher magnifications of the Calcein AM live cell stain revealed that the NCCs had migrated into the stroma (Fig 5B). In transverse sections performed on day 5 after seeding, derived NCCs can be observed on top of the cornea and at the lateral edges where these cells appear to be migrating into the stroma between the collagen fibrils (S5A Fig). Sectioning through the sclera region of the rim showed now cells reside within this region of the tissue (S5B Fig). Taken together, our results suggest that derived NCCs migrate over the sclera to the corneal region of the rim, where they then migrate round the dissected lateral edges and into the exposed collagen fibrils.

**Fig 6:**
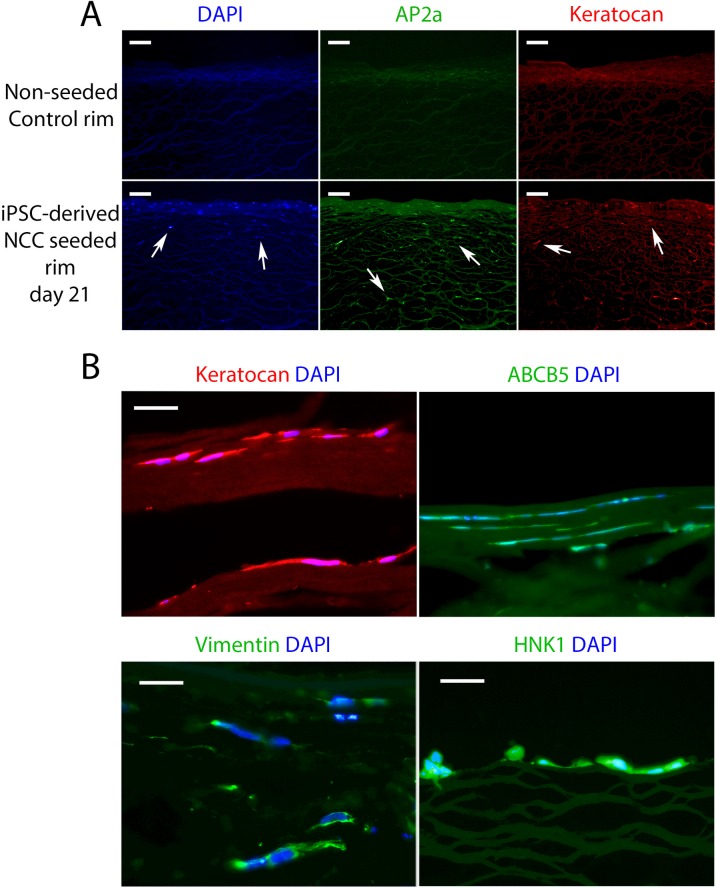
Limbal rim seeded NCCs adopt a corneal keratocyte-like fate and migrate between the collagen lamellae. (A) Control non-seeded rim cryosections showed no DAPI^+^ nuclei or AP2a^+^/ Keratocan^+^ cells. Limbal rims seeded with derived NCCs did contain AP2a^+^/ Keratocan^+^ cells after 21 days of culture (arrows). (B) High magnification images show Keratocan^+^ derived NCCs migrated within the collagen lamellae of the stromal layer of the cornea (this panel is a higher magnification of the Keratocan staining shown in (A). ABCB5^+^, Vimentin^+^ and HNK1^+^ staining was also detected in these cells. Scale bars in (A) represent 100 μm, scale bars in (B) represent 50 μm.

We next wished to observe if hiPSC-derived NCCs cultured on limbal rims acquire a corneal keratocyte-like identity. In transverse sections of control non-seeded corneal rim slices no DAPI, AP2a or Keratocan staining was observed, indicating successful depletion of any endogenous signal (Fig 6A, top panels). DAPI stained nuclei in cells positive for AP2a and Keratocan were observed in limbal rims seeded with hiPSC-derived NCCs (Fig 6A, bottom panels). Higher magnification showed that these cells had migrated in-between the collagen fibrils that constitute the corneal stroma (Fig 6B) and had a similar morphology to human corneal keratocytes found *in vivo* (see S3 Fig and [30]). In addition to Keratocan and AP2a staining, we also observed iPSC-derived NCCs seeded on to corneal rims were positively labelled with ABCB5, Vimentin and HNK1 (Fig 6B). These results suggest that seeding of hiPSC-derived NCCs onto corneal rims promotes expression of corneal keratocyte markers, such as Vimentin, Keratocan and ABCB5, but also maintains NCC-specific marker labelling (HNK1 and AP2a).

**Fig 7:**
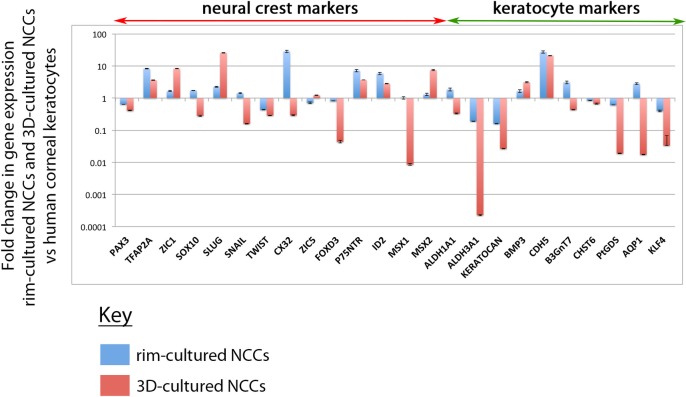
Expression analysis shows limbal rim seeded NCCs adopt an expression profile that is similar to human corneal keratocytes. qPCR analysis indicated hiPSC-derived NCCs cultured on limbal rim slices (blue bars) adopted an expression profile that was more analogous to human corneal keratocytes than hiPSC-derived NCCs grown in 3D cultures (red bars). Importantly, the expression of a number of keratocyte markers was much higher when derived NCCs were cultured on limbal rim slices, such as *ALDH3A1*, *KERATOCAN*, *PtGDS* and *AQP1*.

qPCR analysis of hiPSC-derived NCCs cultured on limbal rim slices for 21 days showed NCC markers such as *PAX3*, *ZIC1*, *SOX10*, *SNAIL*, *TWIST*, *ZIC5*, *FOXD3*, *MSX1* and *MSX2* were similar to levels of expression found in human corneal keratocytes (Fig 7). Only *TFAP2A*, *CX32*, *P75NTR* and *ID2* had levels of expression that were disproportionately higher in the rim-cultured NCCs relative to human corneal keratocytes (Fig 7). Levels of expression for many keratocyte markers were also similar between these two cell populations, especially *ALDH1A1*, *BMP3*, *B3GnT7*, *CHST6*, *PtDGS*, *AQP1* and *KLF4* (Fig 7). *ALDH3A1* and *KERATOCAN* were less highly expressed in hiPSC-derived NCCs cultured on limbal rims than in endogenous corneal keratocytes, and *CDH5* was more highly expressed (Fig 7). Despite these exceptions, the overall gene expression profile of rim-cultured NCCs was more similar than 3D-cultured NCCs to *in vivo* corneal keratocytes (Fig 7).

Taken together, our findings demonstrate that a two-step protocol involving the conversion of hiPSCs to NCCs using the Chambers *et al* (2013) protocol followed by culture on limbal rims is sufficient to induce formation of keratocyte-like cells *in vitro*.

## Discussion

In this study, we have shown hiPSC-derived NCCs adopt corneal keratocyte-like characteristics when cultured in three dimensions or seeded onto cadaveric corneal tissue. Of these two culture methods, we found seeding hiPSC-derived NCCs onto cadaveric human limbal rims was most successful, with these cells adopting a similar morphology and molecular marker profile to native corneal keratocytes after just 21 days of culture.

Other stem cell sources have been utilised for differentiating corneal keratocyte-like cells [31–34], but our study represents the first attempt to generate corneal keratocytes from hiPSCs. A recent report used a similar approach as we present here, by first converting H1 hESCs to NCCs, and then further differentiating these cells in 3D cultures to corneal keratocytes [35]. We believe our protocol is more amenable to downstream applications (including translation to human therapies) as we use a feeder-free system to culture hiPSCs and generate NCCs, whereas the protocol described by Hertsenberg and Funderburgh (2016) uses the PA6 mouse embryonic fibroblast line as feeder cells [35]. In addition, we find seeding corneo-scleral limbal rims with hiPSC-derived NCCs better simulates the *in vivo* environment corneal keratocytes inhabit, and greatly improved the conversion of derived NCCs to a corneal keratocyte-like fate compared to 3D cultures. NCCs seeded onto limbal rims expressed markers such as *KERATOCAN*, *PTGDS*, *AQP1* and *KLF4* at between 10- and 100-fold higher levels compared to 3D-cultured NCCs. *ALDH3A1*, which encodes an aldehyde dehydrogenase important in maintaining the transparency of the corneal stroma [36], was expressed 1000-fold higher in NCCs seeded to limbal rims compared to NCCs cultured in 3D. These differences in expression levels between 3D-cultured and corneal rim seeded hiPSC-derived NCCs are important as they highlight that limbal rim seeded cells are more comparable to native human corneal keratocytes. Our results therefore support a model whereby direct seeding of NCCs to cadaveric corneal tissue effectively promotes corneal keratocyte differentiation. This likely indicates that the corneal stroma contains keratocyte differentiation stimuli despite the removal of endogenous cells. It also suggests that the use of NCCs as a cell-based therapy to repopulate corneal tissue may be a viable clinical option for corneal dystrophies such as keratoconus and should be explored further.

Our study concentrated on looking at the endpoint of NCC differentiation. Follow up studies may benefit from understanding how derived NCCs progressively differentiate into keratocytes and if methods can be modified to improve this process. During embryogenesis, NCC migration and differentiation is closely linked to the extracellular matrix [37]. *In vitro* studies have shown the diameter of matrix fibres acts as a guidance cue and can define neuronal and neural crest differentiation [38, 39]. Given the corneo-scleral rims used here offer a matrix that corneal keratocytes occupy *in vivo*, it is possible that their topographical makeup (such as orientation and diameter of the collagen fibrils) better enables the acquisition of a corneal keratocyte-like fate in our derived NCCs.

Our antibody staining for AP2a showed native corneal keratocytes still express this marker of cranial neural crest, suggesting they retain some level of neural crest fate even in adulthood. Whilst it is difficult to ascertain the developmental time point to which our NCCs correspond relative to *in vivo* embryogenesis, we did find that cells positive for Keratocan are observed after just 21 days. These results support previous findings that corneal keratocytes maintain a level of fate plasticity that is closely matched to the stem cell-like characteristics of NCCs [40]. Thus, our approach of primarily deriving NCCs and then exposing these cells to corneal tissue represents a simple pathway for corneal keratocyte differentiation. Future studies will determine if hiPSC-derived NCCs transplanted into live corneal tissue can aid in recovery from injury or disease. For any clinical or tissue engineering application, it is important to ensure that the cells can be used safely. Whilst we have not analysed cell proliferation in the current study, we did observe extensive proliferation of hiPSCs cultured on limbal rims (data not shown). This same rate of division was not observed when hiPSC-derived NCCs were seeded onto limbal rims, suggesting these cells have a lower proliferative capacity.

In summary, we have established a novel protocol for converting hiPSCs to keratocyte cells. These cells may be of use in future clinical applications, such as cell-based therapies that could treat causes of corneal blindness, such as inherited corneal dystrophies and keratoconus.

## Supporting Information

S1 Fig Characterisation of the successful derivation of NCCs from hiPSCs. AP2a antibody staining showed the NCC derivation protocol used here converted a high percentage of cells to a NCC fate. Scale bar represents 100 μm.
(TIF)

S2 Fig Comparison between Lee *et al* (2010) protocol and Chambers *et al* (2013) protocol for NCC derivation from hiPSCs. Expression of NCC genes *PAX3*, *SOX9*, *SOX10* and *ZIC1* were significantly higher in the Chambers et al (2013) protocol compared to the Lee et al (2010) protocol in our hands.
(TIF)

S3 Fig Non-seeded and non-freeze-thawed human corneas contain Keratocan^+^ and AP2a^+^ keratocytes. Donated cadaveric human cornea cryosections showed endogenous corneal keratocytes can be labelled with antibodies for Keratocan and AP2a. Scale bar represents 100 μm.
(TIF)

S4 Fig Live/Dead cell viability staining shows most limbal rim seeded derived NCCs are viable. Ethidium staining to label dead cells showed minimal cell death at day 4 in cells seeded to limbal rim slices. Scale bar for top panels indicates 200 μm, scale bar for bottom panel indicates 100 μm.
(TIF)

S5 Fig **Sectioning through the sclera shows no cells reside in this region of a seeded rim and derived NCCs likely migrate around the edge of the cornea** (A) Transverse section of cornea stained for DAPI after 7 days of culture with derived NCCs. Arrows indicate the position of derived NCCs on top of the cornea and at the lateral edges where they appear to be entering the collagen fibrils of the stroma. (B) Transverse section view of DAPI (left panel) and ABCB5 (right panel) stained sclera after 21 days of culture with derived NCCs. No cells were observed in any region of the sclera.
(TIF)
