# Supplementary figures and images for: Derivation of Corneal Keratocyte-Like Cells from Human Induced Pluripotent Stem Cells
Source: PLoS One. 2016 Oct 28;11(10):e0165464. doi: 10.1371/journal.pone.0165464 (PMC5085044; doi:10.1371/journal.pone.0165464)

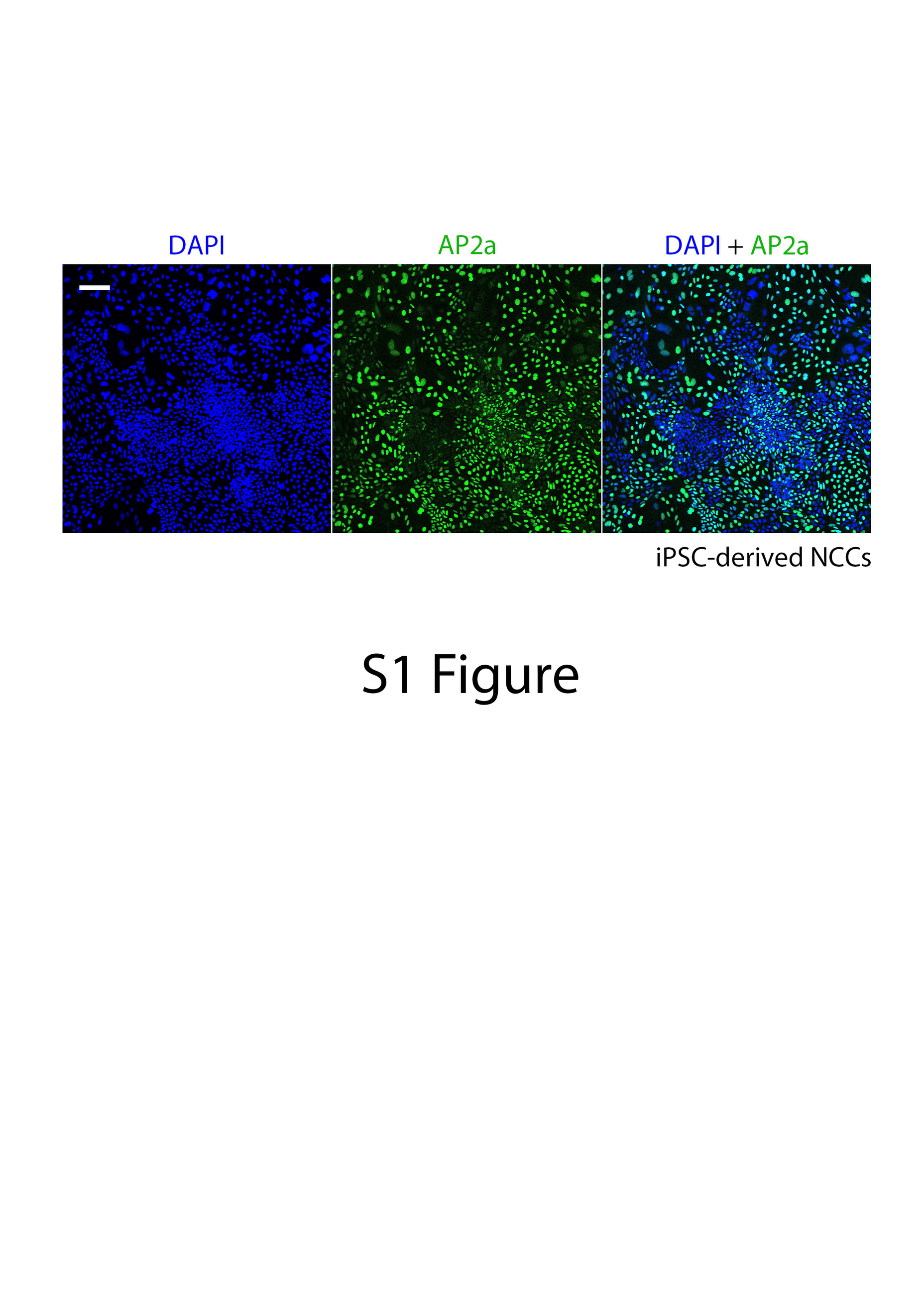

Supplement: S1 Fig — AP2a antibody staining showed the NCC derivation protocol used here converted a high percentage of cells to a NCC fate. Scale bar represents 100 μm. (TIF) [file pone.0165464.s001.tif]

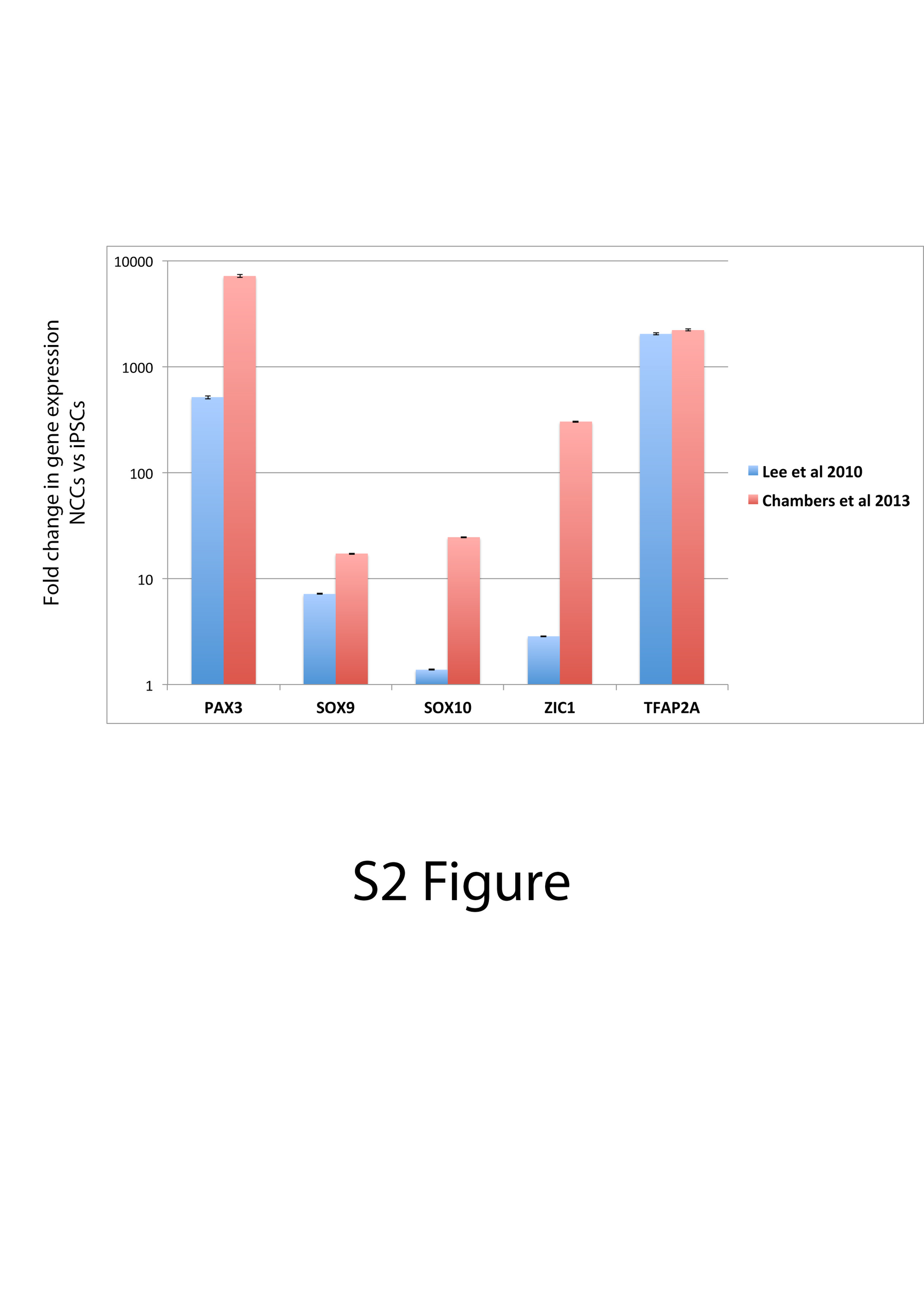

Supplement: S2 Fig — Expression of NCC genes PAX3, SOX9, SOX10 and ZIC1 were significantly higher in the Chambers et al (2013) protocol compared to the Lee et al (2010) protocol in our hands. (TIF) [file pone.0165464.s002.tif]

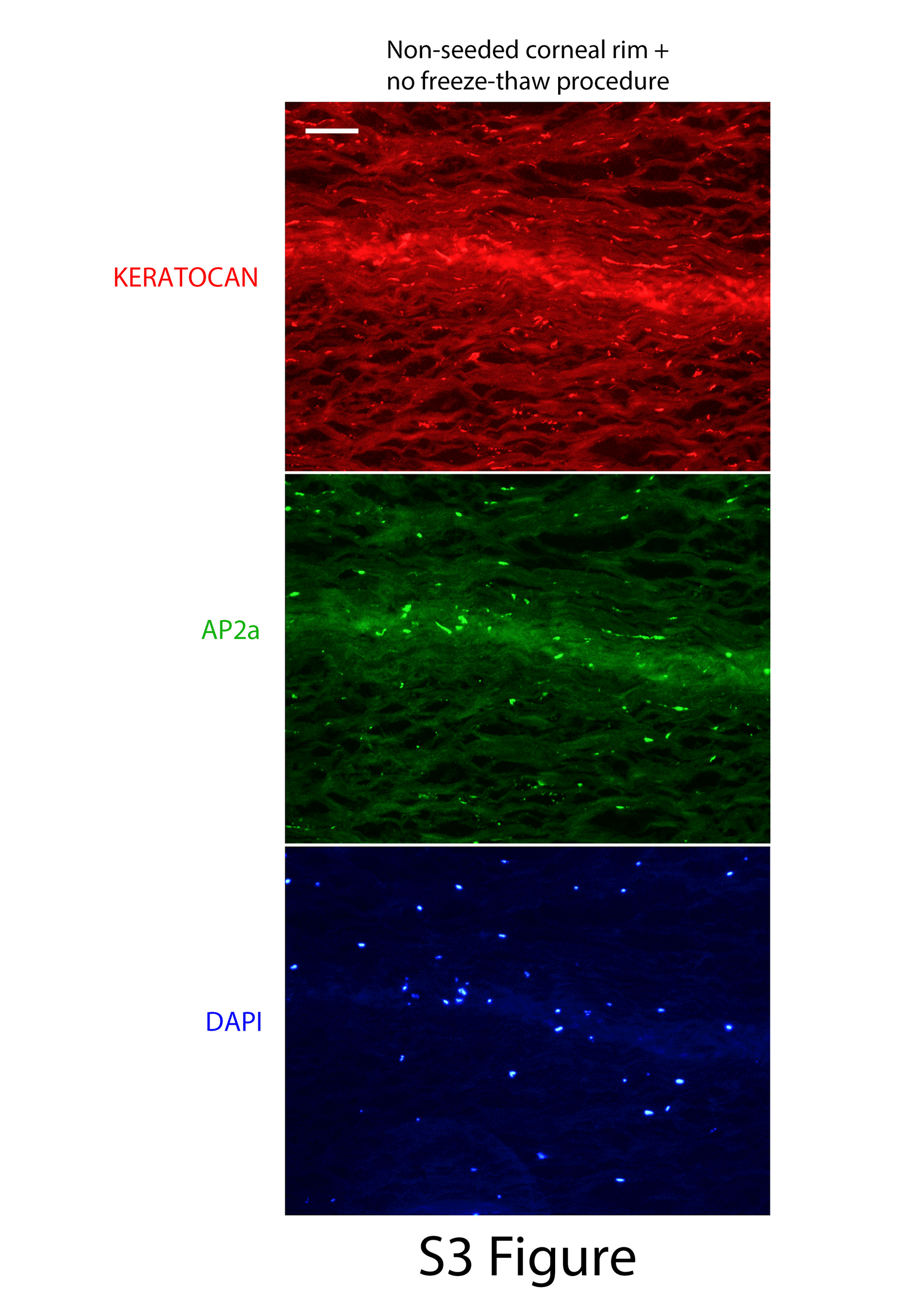

Supplement: S3 Fig — Donated cadaveric human cornea cryosections showed endogenous corneal keratocytes can be labelled with antibodies for Keratocan and AP2a. Scale bar represents 100 μm. (TIF) [file pone.0165464.s003.tif]

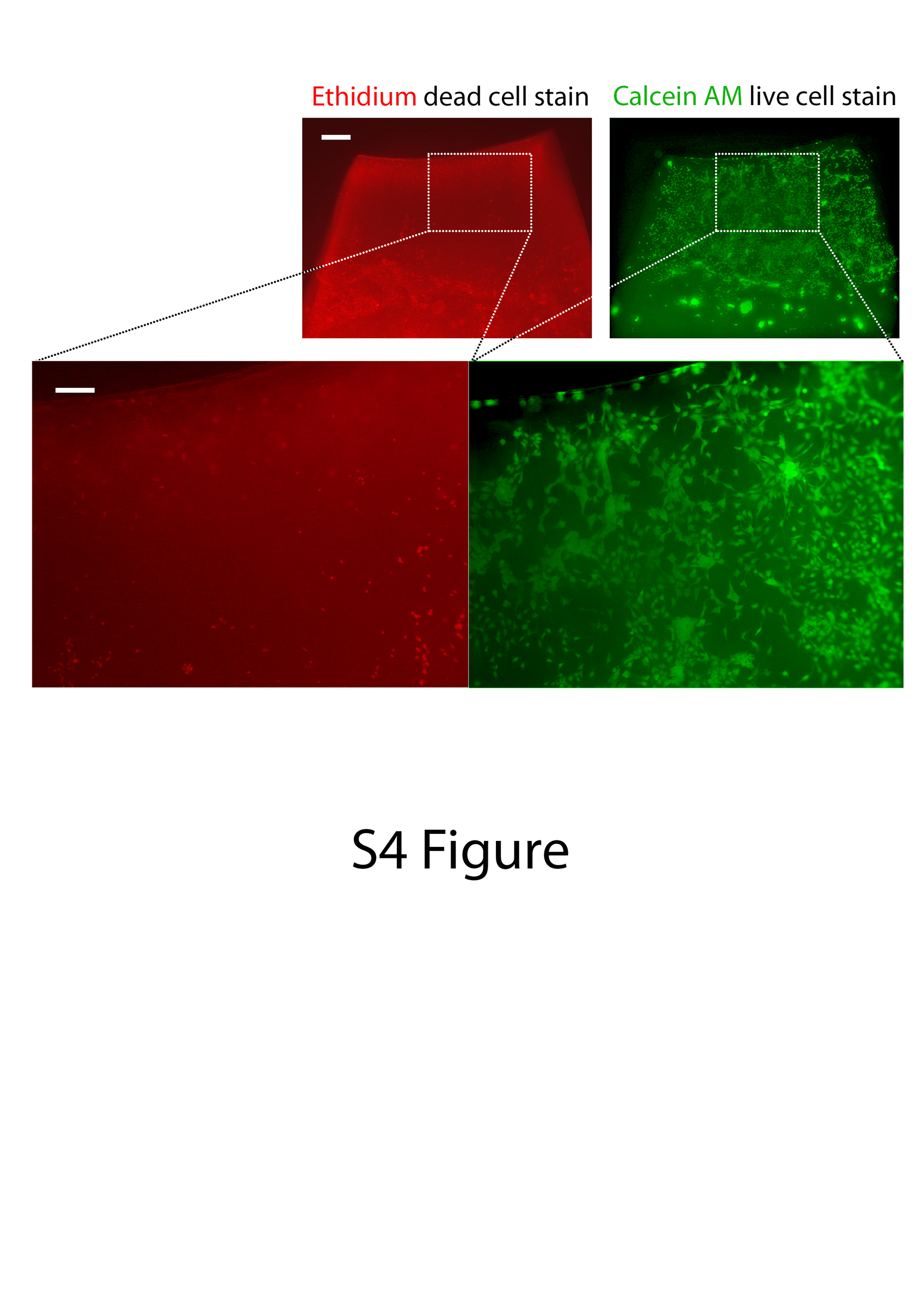

Supplement: S4 Fig — Ethidium staining to label dead cells showed minimal cell death at day 4 in cells seeded to limbal rim slices. Scale bar for top panels indicates 200 μm, scale bar for bottom panel indicates 100 μm. (TIF) [file pone.0165464.s004.tif]

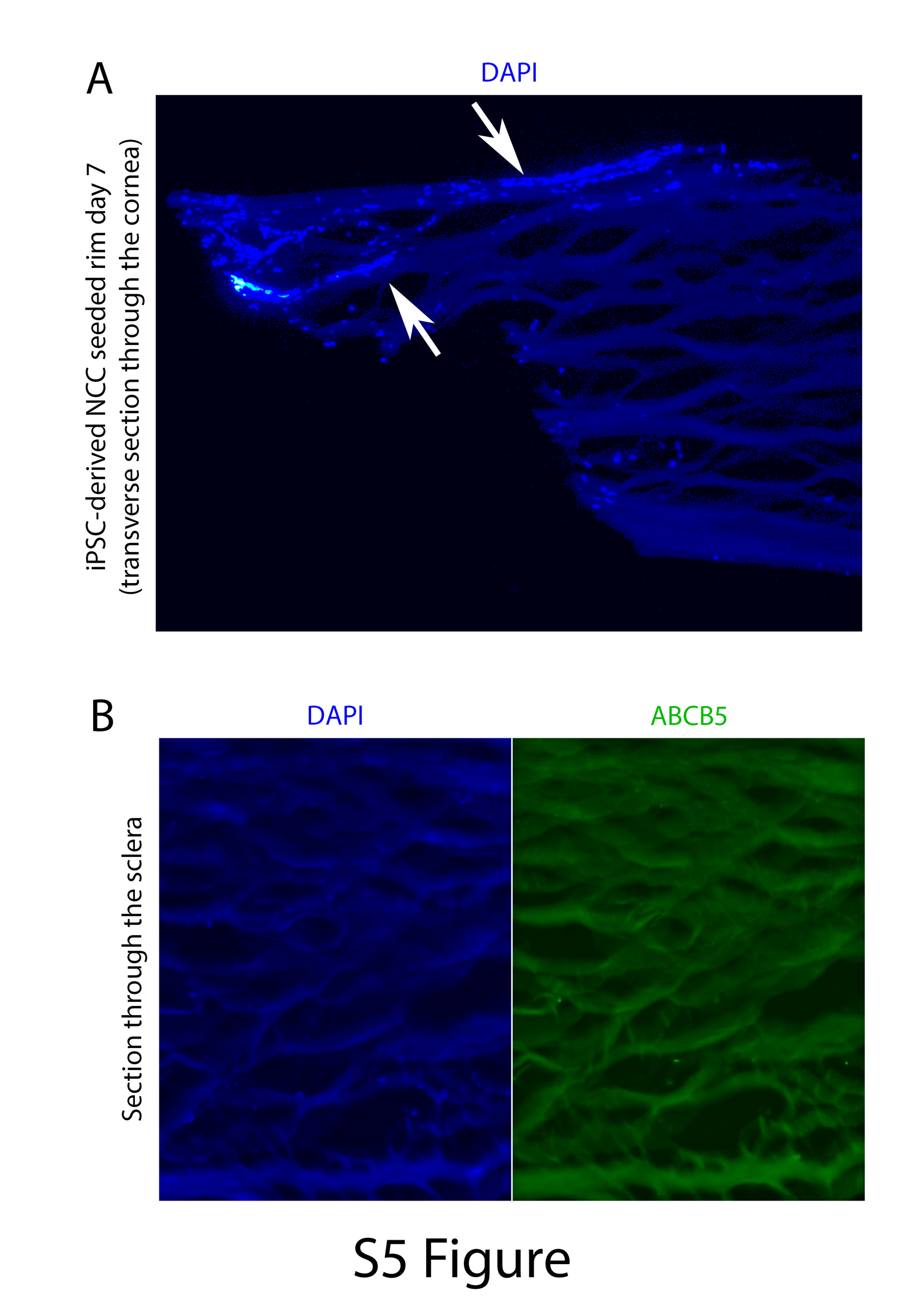

Supplement: S5 Fig — Sectioning through the sclera shows no cells reside in this region of a seeded rim and derived NCCs likely migrate around the edge of the cornea (A) Transverse section of cornea stained for DAPI after 7 days of culture with derived NCCs. Arrows indicate the position of derived NCCs on top of the cornea and at the lateral edges where they appear to be entering the collagen fibrils of the stroma. (B) Transverse section view of DAPI (left panel) and ABCB5 (right panel) stained sclera after 21 days of culture with derived NCCs. No cells were observed in any region of the sclera. (TIF) [file pone.0165464.s005.tif]
